# Feasibility and Preliminary Efficacy of Aerobic Acute Exercise Prior to Immunotherapy and Chemotherapy Infusion in Patients with Metastatic Non-Small Cell Lung Cancer: A Randomized Controlled Trial

**DOI:** 10.3390/jcm15010334

**Published:** 2026-01-01

**Authors:** Manon Gouez, Olivia Pérol, Vincent Pialoux, Virginie Avrillon, Maxime Boussageon, Chantal Decroisette, Lidia Delrieu, Houssein El Hajj, Baptiste Fournier, Romane Gille, Mathilde His, Bénédicte Mastroianni, Aurélie Swalduz, Maurice Pérol, Béatrice Fervers

**Affiliations:** 1 Department of Cancer Prevention and Environment, Léon Bérard Cancer Center, 69008 Lyon, France; 2 Inter-University Laboratory of Human Movement Biology EA7424, University Claude Bernard Lyon 1, University of Lyon, 69622 Villeurbanne, France; 3 1296 Unit Radiations: Defense, Institut National de la Santé et de la Recherche Médicale (INSERM), Health and Environnement, Lyon, France; 4 Department of Medical Oncology, Léon Bérard Cancer Center, 69008 Lyon, France; 5 Residual Tumor & Response to Treatment Laboratory, RT2Lab, Translational Research Department, INSERM, U932 Immunity and Cancer, Institut Curie, Paris, France; 6 IRMES, Institute for Research in Biomedicine and Epidemiology of Sport, UPR7329, Université Paris Cité, Paris, France; 7 INSEP, Institut National du Sport, de l’Expertise et de la Performance, Paris, France

**Keywords:** lung cancer, metastatic, exercise, immunotherapy, immune system

## Abstract

**Background/Objectives:** Recent preclinical studies suggest that acute exercise induces immune modulation, enhances tumor blood perfusion, and is associated with reduced tumor growth. Adding exercise to immunochemotherapy treatment (ICT) has been proposed as a strategy to increase treatment effectiveness. The ERICA trial (NCT04676009) aimed to assess the feasibility of acute aerobic exercise performed immediately before the administration of ICT in patients with metastatic non-small cell lung cancer (mNSCLC) and to explore hypothesis-generating outcomes related to physical fitness and patient-reported outcomes. **Methods:** Newly diagnosed mNSCLC patients were randomly assigned (2:1) to the exercise or control group. The exercise intervention included supervised acute exercise before each of four ICT cycles plus a 3-month home-based walking program with an activity tracker and step goals. The feasibility of the exercise protocol was assessed through adherence, acceptability, tolerability, and safety. Clinical, physical, and patient-reported outcomes were assessed at baseline and after 3 months. **Results:** Twenty-six patients (mean age 60.6 years; SD 10.65) participated, with an 87.5% acceptance rate. In the exercise group (n = 17), 80.9% of participants completed the acute exercise sessions, with a median interval of 38 min [IQR, 20–60] between exercise and ICT. No exercise-related adverse effects were reported. After 3 months, 60% of participants in the exercise group were classified as active and maintained their step goals. Self-reported measures suggest that maintaining physical fitness is favorable for reducing fatigue and insomnia, and therefore improving quality of life. **Conclusions:** Acute exercise performed immediately before each ICT administration in patients with mNSCLC appears feasible and safe.

## 1. Introduction

Lung cancer is the second most diagnosed cancer, accounting for 11.6% of total cancer cases, with an estimated 2.2 million new cases in 2020. It is the leading cause of cancer-related deaths, contributing to 18.4% of all cancer deaths [1]. Non-small cell lung cancer (NSCLC) represents 80 to 90% of all lung cancer cases, and more than half of all lung cancer cases are diagnosed at an advanced stage because of its paucity of symptom, resulting in poor survival rates [2,3].

Advances in treatment, particularly the combination of platinum-based chemotherapy (CT) with immune checkpoint inhibitors (ICPi) targeting programmed cell death 1 (PD-1) or programmed death ligand 1 (PD-L1), have improved survival in advanced NSCLC patients [4]. However, many patients exhibit primary resistance, and even initial responders often relapse due to acquired resistance [5]. ICPi efficacy depends on effective antitumor immune response, including adequate immune system competence, tumor infiltration by T lymphocytes, recognition of tumor-specific antigens, and a permissive tumor microenvironment [6,7]. In this context, physical activity (PA) after cancer diagnosis has been associated with improved cancer-specific and overall survival in observational studies [8], as well as improved quality of life, fatigue, and cardiorespiratory fitness [9]. Exercise has been shown to exert immunomodulatory effects [10]. A single exercise bout induces lymphocytosis and mobilizes immune cells [11,12,13,14]. Preclinical studies further suggest that exercise-induced immune cell mobilization [15,16,17] and increased tumor blood perfusion during exercise are associated with reduced tumor growth [18]. Emerging studies indicate that exercise may modulate systemic and local immune responses, potentially altering tumor immune phenotype and influencing immunotherapy response [19].

Although preclinical and observational data suggest that exercise may modulate immune function, clinical evidence in patients receiving ICT remains limited. In particular, the feasibility and safety of acute exercise performed immediately before treatment administration have not been evaluated in patients with mNSCLC. Given the advanced disease stage, symptom burden, and treatment-related toxicities in this population, establishing feasibility is a necessary first step before exploring potential clinical effects. Therefore, the primary aim of this study was to assess the feasibility of repeated supervised acute exercise sessions performed immediately prior to first-line immunochemotherapy in patients with metastatic NSCLC [20].

### Objectives

This study aimed to assess the feasibility of repeated supervised acute exercise sessions before each of the four first-line ICT cycles (pembrolizumab with pemetrexed or paclitaxel and cisplatin or carboplatin) in mNSCLC patients. To maintain physical fitness and ensure engagement, the intervention was combined with a home-based walking program. The primary feasibility endpoints were adherence, acceptability, tolerability, and safety of the exercise protocol. The secondary endpoints evaluated preliminary effects on physical fitness, physical activity level, patient-reported outcomes, body composition, treatment completion rates and severe treatment toxicities.

## 2. Materials and Methods

### 2.1. Study Design

The design and methods of the ERICA (Exercise inteReaction Immunotherapy Chemotherapy and cAncer) trial have been previously described [20]. This prospective, single-center, 2:1 randomized controlled, open-label feasibility study was conducted from January 2021 to June 2023 at the Léon Bérard Comprehensive Cancer Centre (CLB), Lyon, France. The protocol was approved by the French ethics committee (N°ID-RCB 20.09.04.65226, 8 December 2020), reported to the National Commission for Data Protection and Liberties (CNIL; ref:1994192), and registered at ClinicalTrials.gov (NCT04676009). The study was conducted in accordance with the Helsinki Declaration. All participants provided written informed consent.

### 2.2. Participants and Eligibility Criteria

Eligible patients were aged ≥ 18 and <80 years, who were considered (according to the multidisciplinary tumor board decision) and treated as metastatic NSCLC without *EGFR* mutation or *ALK* rearrangement and were eligible for first-line immunochemotherapy with pembrolizumab. They were required a WHO performance status < 2 (PS), the ability to engage in PA, and a medical certificate confirming no contraindication to exercise [20].

The exclusion criteria included the following: bone metastases with fracture risk, history of fractures or unconsolidated pathological fractures; history or coexistence of other primary cancers (except in situ and/or basal cell carcinoma and/or non lung cancer in remission > 5 years); history or risk of cardiovascular disease; stage IV chronic obstructive pulmonary disease; severe undernutrition defined according to the French National Authority for Health [21]; severe anemia; uncontrolled type 2 diabetes; physical or mental disabilities precluding exercise; and inability to read and understand French.

### 2.3. Patient Screening and Baseline Evaluation

Patients were systematically screened during weekly lung cancer multidisciplinary board meetings. Eligible patients were introduced to the study during a pretreatment consultation, during which the oncologist explained the objectives and protocol. After providing informed consent, patients underwent clinical screening, including (1) PS assessment and blood pressure, (2) echocardiography and electrocardiogram performed by a cardiologist and (3) glycated hemoglobin measurement for diabetic patients. Patients meeting the eligibility criteria were then included in the study (D0).

### 2.4. Randomization and Group Allocation

At baseline (D0), patients were randomly assigned at a 2:1 ratio to either (i) the exercise group, which received PA and nutrition recommendations, supervised acute physical exercise before each ICT infusion and a home-based unsupervised walking program with an activity tracker; or (ii) the control group, which received PA and nutrition recommendations only. Randomization was stratified using a dynamic minimization algorithm based on the two following factors: sex (male vs. female) and tumor histology (squamous vs. non-squamous).

### 2.5. Intervention

All participants received standardized PA and nutrition recommendations, as previously described [20]. The exercise intervention included supervised acute exercise immediately before each ICT infusion, with intensity individualized based on submaximal cardiorespiratory fitness assessments [20]. Each session included a 5-min warm-up on a cycle ergometer at 40% of the first ventilation threshold (VT1), followed by 30 min of moderate interval training (5 × 3 min bouts at 100–110% VT1, interspersed with 3-min low-intensity active recovery periods at 60% VT1). Sessions ended 15 min before treatment infusion. Heart rate (HR), load, revolutions per minute (RPM), dyspnea and perceived exertion (Borg scale) were monitored by an exercise physiologist. The 3-month home-based walking program targeted ≥6000 steps per day between treatment cycles, tracked via a Fitbit Inspire.

### 2.6. Standard Treatment for Metastatic Non-Small Cell Lung Cancer

All participants received standard ICT, consisting of four 3-week cycles of pembrolizumab combined with platinum-based CT, followed by pembrolizumab maintenance for squamous cell carcinoma or pembrolizumab plus pemetrexed maintenance for non-squamous cell carcinoma. Each cycle included pembrolizumab (200 mg flat dose), carboplatin (AUC 5) or cisplatin (75 mg/m^2^) plus pemetrexed (500 mg/m^2^) with B9-B12 supplementation for non-squamous cell carcinoma or carboplatin (AUC 6) plus paclitaxel (200 mg/m^2^) for squamous cell carcinoma. Induction treatment lasted approximately three months before maintenance treatment began.

### 2.7. Data Collection

Clinical, physical, and patient-reported outcomes were assessed at baseline (D0) and after the 3-month intervention period (M3). Psychometric follow-up was conducted at 6 months (M6). Survival was monitored in January 2024 using electronic medical records.

### 2.8. Outcome Measures

#### 2.8.1. Primary Endpoint: Feasibility of the Intervention

Feasibility was assessed through adherence rates, which were calculated as the proportion of completed to planned exercise sessions.

Adherence to the home-based walking program was assessed by the proportion of patients achieving the daily target of 6000 steps recorded by the activity tracker.

Acceptability (percentage of eligible patients who agreed to participate) and attrition (percentage of patients who withdrew consent or were lost to follow-up due to symptom burden, death, or other reasons, excluding treatment changes due to disease progression) were also measured.

Tolerability metrics included exercise dose modification, rates of training interruption or permanent discontinuation [22]. Exercise dose modification was defined as any reduction in session duration or intensity, whereas permanent discontinuation referred to complete withdrawal from the intervention, regardless of whether patients remained in the study.

Safety was evaluated by tracking serious and nonserious adverse events (AEs) during supervised exercise sessions.

#### 2.8.2. Secondary Endpoints

Secondary endpoints assessed preliminary intervention effects through repeated measures at D0 and M3, including the following:

- Physical fitness: submaximal oxygen consumption and upper- and lower-body muscular strength;
- Physical activity level: assessed by the Godin Leisure Time Physical Activity Questionnaire (GLTAPQ) (active ≥ 14 kcal/kg/week; moderately active: 7–13.9 kcal/kg/week, insufficiently active: <7 kcal/kg/week [23]);
- Body composition was assessed by the skeletal muscle index (SMI) at the third lumbar vertebra (L3), skeletal muscle density (SMD), lean body mass (LBM), visceral adipose tissue (VAT) and subcutaneous adipose tissue (SAT). The images were independently reviewed by two evaluators (technicians and physicians) blinded to group allocation, who used 3D-Slicer to assess the sarcopenic status of the participants. The diagnosis of sarcopenia was defined on the basis of L3 SMI cut-off values < 43.0 cm^2^/m^2^ for men with a BMI < 25.0 kg/m^2^, <53.0 cm^2^/m^2^ for men with a BMI ≥ 25.0 kg/m^2^, and <41.0 cm^2^/m^2^ for women [24];
- Patients reported outcomes (at D0, M3 and M6): health-related quality of life (EORTC QLQ C30, LC13), cancer-related fatigue (QLQ FA-12), and sleep quality (Insomnia Severity Index).

Treatment completion rates and severe treatment toxicities (grade ≥ 3) were recorded using the National Cancer Institute’s Common Terminology Criteria for Adverse Events (NCI-CTCAE) V.5.0.

The detailed description of outcome measures and measurement procedures has been previously published in the study protocol by Gouez et al. 2022 [20].

### 2.9. Statistical Analyses

As the primary endpoint was feasibility, no formal sample size calculation was performed. Feasibility trials typically recommend sample sizes between 24 and 50 [25]. This study included 30 participants (20 in the exercise group and 10 in the control group), whose data were determined pragmatically and aligned with published recommendations [26].

The participants’ characteristics and outcomes were summarized via means (±SD or 95% CI) and medians (IQRs) for quantitative data and frequencies and percentages for qualitative data, with missing data reported. Baseline characteristics and outcomes were compared between groups using nonparametric ANOVA of ranked data because of the small sample size.

Changes from baseline (D0) to the 3-month follow-up (M3) were analyzed using the Wilcoxon signed-rank test for continuous variables (aerobic fitness, muscle strength, patient-reported outcomes (PROs), and body composition parameters), which were non-normally distributed.

Categorical outcomes, such as the presence of sarcopenia, were compared using a chi-square test at D0 and M3. Paired categorical data (e.g., sarcopenia progression) were analyzed using the McNemar and Fisher tests.

A *p*-value < 0.05 was considered statistically significant. Given its exploratory nature, no adjustments were performed in this feasibility study. Analyses were performed using R software (v4.2.2).

## 3. Results

### 3.1. Recruitment

**Figure 1:**
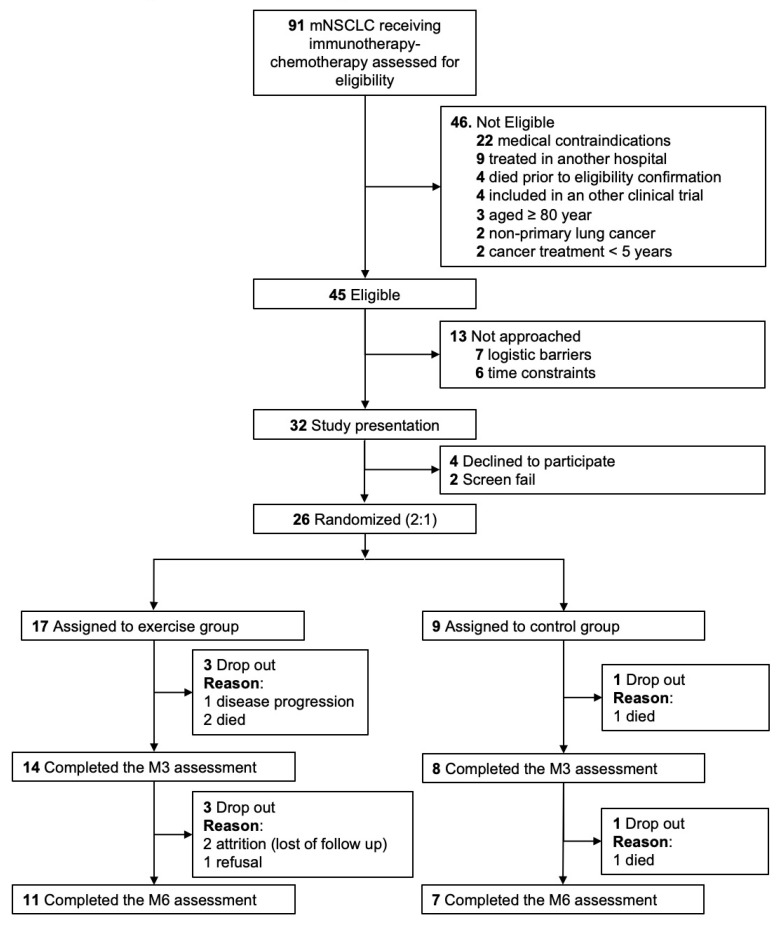
CONSORT flow diagram of the ERICA randomized controlled trial.

From January 2021 to June 2023, 91 patients were screened, of whom 45 (49.5%) met the eligibility criteria (Figure 1). Medical contraindications were the main reason for ineligibility. Among the 45 eligible patients, the study was not presented to 13 patients due to logistical and organizational barriers (i.e., the time to treatment initiation was too short to complete clinical screening and baseline assessments before treatment start (n = 6), and distance from the cancer center prevented re-attendance for assessments (n = 7)). Of the 32 patients to whom the study was presented, 4 declined to participate, resulting in an acceptance rate of 87.5%, and 2 were erroneously included despite medical contraindications (pulmonary embolism or ischemic heart disease). The remaining 26 patients were randomized: 17 to the exercise group and 9 to the control group (Figure 1).

During the intervention, two patients were excluded due to treatment changes resulting from disease progression (one per arm). The attrition rate for the exercise group was 11.8% (2/17), with no attrition in the control group. Four participants died of mNSCLC (2 per arm), and one patient discontinued the study due to disease progression (Figure 1).

#### Baseline Characteristics

Table 1 summarizes the baseline characteristics of the patients. The majority were men (72.0%), with a mean age at diagnosis of 60.6 years (±10.5) and an average body mass index (BMI) of 24.1 kg/m^2^ (±4.1) (Table 1).

A history of smoking was present in 80.8% of the participants, 52.2% had cardiovascular disease, 34.8% had a history of thoracic radiotherapy, and 30.4% had a history of metabolic disorders. The most common regimen was carboplatin/pemetrexed/pembrolizumab (65.9%), followed by cisplatin/pemetrexed/pembrolizumab (26.9%, exclusively in the exercise group) and carboplatin/paclitaxel/pembrolizumab (7.7%).

At baseline, 59.7% were insufficiently active, 19.3% were moderately active, and 15.4% were active. The mean VT1 oxygen consumption was 15.50 ± 3.96 mL/min/kg (Table 1).

### 3.2. Primary Endpoints

#### 3.2.1. Adherence to the Acute Exercise Session

**Figure 2:**
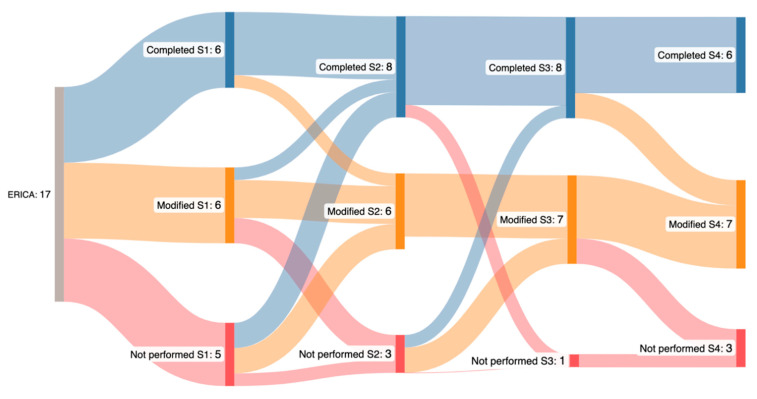
Evolution of adherence to acute exercise sessions before ICT administration, i.e., completed as scheduled (in blue), modified (duration and/or intensity; in yellow), or not performed (in red). S1, S2, S3 and S4 = acute exercise session before ICT cycles 1, 2, 3 and 4, respectively.

The mean adherence rate was 80.8%, with a median of 4 sessions [IQR, 3–4], and 52.9% of the participants completed all four sessions. The completion rates ranged from 70.5% to 93.8% (Figure 2).

The median interval from the end of the exercise to the start of ICT was 38 min [IQR, 20–60], varying by cycle (i.e., 33, 40, 28 and 57 min for the first, second, third, and fourth ICT cycles, respectively) (Supplementary Information (SI) 1, SI.1). Non-completion was primarily due to pain (n = 3), surgery (n = 1), or COVID-19 (n = 1).

#### 3.2.2. Tolerability and Safety of the Acute Session

Only 17.7% of the participants completed all sessions as prescribed. Exercise dose adjustments (intensity and/or duration) were required for 58.8% of the participants, accounting for 48.1% of the total sessions (Table 2). No serious exercise-related AEs were reported.

#### 3.2.3. Adherence to the Home-Based Walking Program

**Figure 3:**
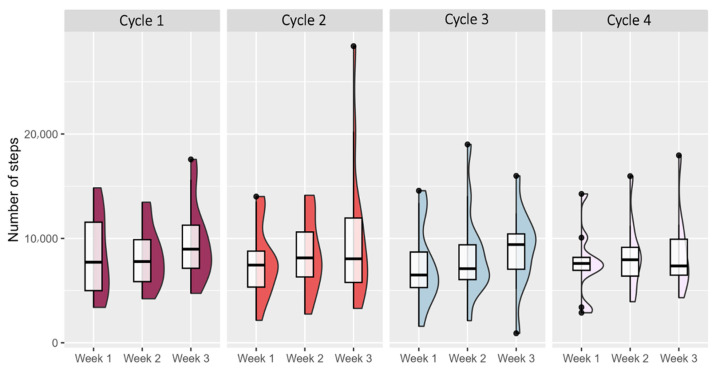
Boxplots showing the distribution of daily steps per week between treatment cycles in the exercise group. Data are presented as the median (IQR).

The exercise group maintained a median daily step count of 8550 (SI.1). Compliance increased from 58% in the first cycle to 84% in the last cycle, although 23.5% to 47.1% of the step count data were missing in some weeks. Step counts followed a cyclical pattern, decreasing after treatment and increasing in the third week of each cycle (Figure 3).

### 3.3. Secondary Endpoints

#### 3.3.1. Physical Fitness

**Figure 4:**
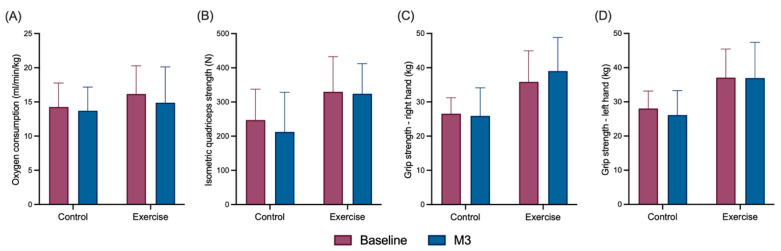
Comparison of physical fitness between groups at baseline and M3. Histograms showing changes in oxygen consumption at VT1 (**A**), isometric quadriceps strength (**B**) and handgrip strength (**C**,**D**) maximum in the exercise and control group. The bars represent the means, and the error lines indicate the standard deviation. M3: Month3.

From baseline to M3, no meaningful between-group differences were observed in physical fitness outcomes (Figure 4). All estimated changes with 95% confidence intervals are presented in detail in the SI.7.

#### 3.3.2. Physical Activity Level

After 3 months, 45.4% of the participants in the exercise group were classified as “Active” whereas 43.3% of the participants were previously “Insufficiently Active” at baseline. The mean global Leisure-Time PA score increased from 13.8 (±14.5) to 30.9 (±14.8) in the exercise group, with a within-group change of 15.3 (95% CI: 0.24 to 30.37). In the control group, the score increased from 16.9 (±15.9) to 23.4 (±30.5), corresponding to a within-group change of 7.0 (95% CI: −25.3 to 39.3). The estimated between-group difference in change was 8.3 (95% CI: −25.5 to 42.2), indicating no between-group effect (Table 2). Categorical analysis showed minor changes in the control group, the proportion of active individuals decreased by 9.7%, moderately active individuals increased by 27.8%, and insufficiently active/sedentary individuals decreased by 18.1% (Table 2, SI.7).

#### 3.3.3. Body Composition Assessment and Sarcopenia

In the exercise group (n = 17), a significant decrease in the skeletal muscle index (SMI) was observed between baseline and the 3-month follow-up (M3) (49.2 ± 6.16 cm^2^/m^2^ vs. 46.2 ± 7.26 cm^2^/m^2^, *p* = 0.02) (Table 2). In both groups, the lean body mass (LBM) tended to decrease between baseline and M3 but did not reach statistical significance. According to the L3 SMI cut-off values, the proportion of participants classified as sarcopenic increased by 26.9% in the exercise group, whereas in the control group, it increased by only 11.2% (after M3). There was no significant association between weight change (gain, loss, or stability) and sarcopenia change (development, improvement, or stability) in either group (Table 2, SI (Supplementary Information) SI.2 and SI (Supplementary Information) SI.3).

#### 3.3.4. Patient-Reported Outcomes

*Changes in patient-reported outcomes from D0 to M3*

There was a non-significant trend towards improved global health status in the exercise group (+5.77, 95% CI: −6.97, 18.51), whereas the control group experienced a non-significant decline (−11.46, 95% CI: −29.29, 6.38) (SI.4).

Overall, physical, emotional, and cognitive fatigue appeared to be lower and had decreased in the exercise group, whereas the control group had higher scores and an increase after three months of study, suggesting a worsening of fatigue. Emotional fatigue increased significantly in the control group (+12.50, 95% CI: 0.92, 24.08), and the changes between inclusion and M3 differed significantly between the groups (−15.92, 95% CI: −31.2. −0.64). The global score of the insomnia severity index decreased significantly by 30.0% between D0 and M3 in the exercise group (−4.67, 95% CI: −7.96, −1.38), indicating an improvement in sleep quality (SI.4).

*Changes in patient-reported outcomes after 6 months of follow-up*

In the exercise group, the initial improvement in the global health score observed after 3 months of intervention returned to baseline levels by 6 months. Although none of the changes reached statistical significance, there was a notable decrease in PRO scores within the exercise group after 6 months of follow-up (SI.5). The control group showed an improvement in scores between the 3-month and 6-month assessments.

#### 3.3.5. Treatment Completion and Adverse Events

The exercise group experienced three grade 3 and one grade 4 treatment-emergent adverse event (TEAEs), including anemia, anorexia, and increased troponin I. The control group had eleven grade 3 and two grade 4 TEAEs, such as anemia, anorexia, aplasia, asthenia, neutropenia, thrombocytopenia, weight loss, diarrhea, and increased troponin I. Notably, 57.1% of these AEs occurred in the same participants in the control group (SI.6).

Over the 3-month period, 31.6% of the control group experienced a dose reduction during ICT, whereas 19.8% of the exercise group experienced a dose reduction (SI.6). Four participants discontinued ICT before completing 4 cycles, three from the exercise group (deaths, n = 2; disease progression, n = 1) and one from the control group (death). Additionally, one participant in the control group died between M3 and M6.

At 3 months, 14 participants (53.9%) achieved a partial response to treatment, with 62.5% in the exercise group and 44.4% in the control group. Seven participants (28.0%) had stable disease, with 18.7% in the exercise group and 44.4% in the control group (SI.6).

## 4. Discussion

ERICA is the first study to assess the feasibility of supervised acute exercise performed immediately before ICT administration in patients with mNSCLC and, more broadly, within oncology. High acceptance, strong adherence, low attrition, and the absence of adverse events support the feasibility of this innovative intervention. Exploratory analyses suggest potential benefits on patient-reported outcomes, physical activity levels, and maintenance of physical fitness levels.

### 4.1. Innovative Integration of Acute Exercise Prior to Immunochemotherapy Administration in Patients with Metastatic Lung Cancer

Most existing evidence on exercise in oncology comes from early-stage settings, either after treatment or between chemotherapy sessions. Only two small studies have explored low-intensity exercise during chemotherapy infusion, both showing feasibility without adverse events in early-stage breast and ovarian cancer patients [27,28]. Kerrigan et al. reported that early-stage breast cancer patients could exercise for 16.4 min at 30–40% of their heart rate reserve, with fewer side effects than non-exercisers [27]. Thomas et al. reported 100% adherence to two supervised 20-min low-intensity cycling sessions during CT in early-stage breast and ovarian cancer patients [28]. The ERICA trial extends these findings to a metastatic lung cancer population by evaluating exercise performed immediately prior to immune-chemotherapy administration, with high adherence and no safety concerns.

Despite American College of Sports Medicine (ACSM) recommendations for PA during cancer treatment [29], metastatic patients remain underrepresented in exercise trials [30]. The high acceptance observed in the ERICA trial, together with results from trials such as PREFERABLE-EFFECT (n = 357) and ABLE (n = 49) in metastatic breast cancer [31,32] support the feasibility of implementing structured exercise interventions in advanced disease. These findings reinforce the relevance of integrating exercise as part of comprehensive supportive care in metastatic cancer.

### 4.2. Preliminary Effects of the Intervention on Physical Activity Level, Physical Fitness and PROs in Patients with Metastatic NSCLC

Although the acute exercise intervention combined with the home-based walking program did not significantly improve the physical fitness of participants in the exercise group at the end of the intervention, patients reported an increase in PA level, as reflected by the GLTPA score. While a recent study reported a median decrease of 12.5% in daily step counts among patients with metastatic NSCLC [33], participants in the ERICA exercise group maintained relatively high levels of physical activity, with an average of 8550 steps per day among those with available data. According to the Tudor-Locke and Bassett graduated step index for categorizing pedometer-determined habitual PA adults, ERICA participants were classified as “somewhat active,” averaging between 7500 and 9999 steps per day [34].

Step count data suggested a cyclical pattern, with a decrease during the week following each treatment cycle and a rebound during the third week post-treatment. However, step count data were incomplete for several participants, with missingness occurring more frequently in the later weeks of the program and sometimes related to treatment toxicity or disease progression. As a result, adherence to the home-based walking program may be overestimated. These findings also highlight that step count diaries can represent an additional burden for patients with metastatic disease. Regular visits every three weeks allowed review of recent walking activity and may have contributed to maintaining motivation throughout the intervention. The ERICA trial support strategy, combining step targets, activity tracking, and scheduled follow-up visits, may therefore help sustain PA levels between treatment cycles, although future studies should consider less burdensome or automated monitoring approaches.

Currently, data on the effects of PA programs on physical fitness in patients with NSCLC remain limited and inconclusive. A meta-analysis by Peddle-McIntyre et al. (2017), including six trials and 221 participants with advanced-stage lung cancer, reported a significant improvement in physical fitness, measured by the six-minute walk test, in intervention groups at the end of the programs [35]. However, only one study demonstrated a significant increase in VO_2_peak following an eight-week high- to moderate-intensity interval training program [36]. In the ERICA trial, VO_2_ values at VT1 (16.16 ± 4.12 vs. 15.1 ± 3.4) were comparable to those reported by Hwang et al. (2012) [36] in their assessment of VO_2_peak. Compared with our intervention, their more intensive program—three 30–45-min cycling sessions per week—resulted in significant improvements in VO_2_peak, suggesting that the dose of acute exercise combined with a home-based walking program in ERICA may have been insufficient to induce measurable gains in physical fitness.

While growing evidence highlights the importance of body composition for cancer outcomes and response to immunotherapy [37], our intervention did not lead to improvements in body composition. Notably, a decrease in skeletal muscle index was observed in the exercise group, representing a clinically relevant and unexpected finding. This may reflect an insufficient anabolic stimulus, as the intervention was not designed to target muscle hypertrophy and did not include resistance training or nutritional optimization. Additional contributing factors may include more adverse baseline clinical characteristics in the exercise group and the small sample size, which limits the precision of estimates. Given the established association between sarcopenia, systemic inflammation, and reduced efficacy of PD-1 inhibitors in NSCLC [38], these findings underscore the need for future interventions specifically designed to preserve or increase muscle mass. Multimodal rehabilitation programs combining resistance training and targeted nutritional support may represent a more appropriate strategy to counteract muscle loss in this population.

The ERICA study revealed a trend toward improvement in the global quality-of-life score in the intervention group compared with the control group at 3 months. These findings are in line with recent studies in mNSCLC patients, which have reported improvements in disease-specific health-related quality of life following exercise interventions [35,39,40,41]. In addition, physical, emotional, and cognitive fatigue appeared to be lower and to decrease over the 3-month intervention period in the exercise group, whereas a trend toward increased fatigue was observed in the control group. However, patients in the exercise group received more frequent supervision and contact than those in the control group, which may have contributed to patient-reported outcomes through attention and expectation effects. Accordingly, improvements in quality of life and fatigue may reflect, at least in part, the combined effects of the exercise intervention and the enhanced support and engagement provided.

### 4.3. Strengths and Limitations of the ERICA Study

The ERICA study has several strengths. This study was conducted in a homogeneous population of mNSCLC patients treated with ICT combination, allowing for a better understanding of exercise feasibility in this population, with 60% of participants in the exercise group presenting with a history of cardiovascular disease and 35% with previous thoracic radiotherapy. The supervision and rigorous control of exercise dosage and close collaboration with healthcare personnel ensured consistent program follow-up and integration into the overall treatment plan. Finally, rigorous safety measures, including echocardiographic assessments, electrocardiograms, and nutritional evaluations, ensured participant safety throughout the intervention.

While the ERICA study provides the foundation for larger, more comprehensive studies to assess the impact of exercise interventions, it has certain inherent limitations that offer opportunities for future research. The main limitation is the small sample size, which restricts statistical power and limits the generalizability of the findings. From a methodological perspective, analyses were exploratory, unadjusted, and at risk of false positives due to multiple outcomes. Due to logistical constraints, particularly time constraints related to treatment initiation, six eligible patients could not be enrolled, and in four cases, baseline physical fitness assessments were replaced by data collected during the first exercise session. Although the median interval between the end of the exercise session and immunochemotherapy administration was 38 min, supporting the feasibility of acute exercise prior to treatment, this delay exceeded 60 min in 31% of sessions, highlighting organizational challenges in real-world settings. Moreover, this was a single-center study conducted within the specific procedures and timelines of the CLB Cancer Center, and the transferability of the protocol to other healthcare settings warrants further evaluation.

## 5. Conclusions

The ERICA study demonstrated the feasibility of acute exercise performed immediately before ICT administration in patients with mNSCLC. No exercise-related AEs were reported, and there was no attrition during the intervention period. Although this study of a limited sample size did not show improvements in the exploratory parameters assessed, the feasibility objective of the intervention was largely validated. These data are valuable for the implementation of a multicenter randomized trial to further investigate the effects of the intervention on clinical, physical, and psychosocial parameters. Future analyses of the evolution of immune biomarkers will contribute to a better understanding of the effects of the acute exercise intervention.

## Figures and Tables

**Table 1:** Sociodemographic and clinical characteristics of the ERICA study population at baseline.

	Overall (n = 26)	Exercise Group (n = 17)	Control Group (n = 9)
Age, mean (SD)	60.62 (10.65)	60.11 (10.93)	61.57 (10.68)
Gender, n (%)			
Women	8 (30.77)	4 (23.53)	4 (44.44)
Men	18 (69.23)	13 (76.50)	5 (55.55)
Living situation, n (%)			
Coliving	15 (57.69)	11 (64.71)	4 (44.44)
Living alone	11 (42.31)	6 (35.29)	5 (55.56)
Social vulnerability score, n (%)			
Absence of social deprivation (<30)	9 (36.00)	6 (37.50)	3 (33.30)
Social deprivation (≥30)	16 (64.00)	10 (62.50)	6 (66.70)
Missing	1 (3.85)	1 (5.88)	-
Smoking Status, n (%)			
Current smoker	3 (11.54)	2 (11.76)	1 (11.11)
Former smoker	18 (69.23)	12 (70.59)	6 (66.67)
Never smoker	5 (19.23)	3 (17.65)	2 (22.22)
Packs-Years of tobacco, mean (SD)	31 (14.06)	31 (15.56)	31 (11.58)
Alcohol intake, n (%)	11 (42.31)	7 (41.20)	4 (44.44)
Medical history, n (%)			
Cardiovascular disease	12 (52.17)	9 (60.00)	3 (37.50)
Thoracic radiotherapy	8 (34.78)	6 (40.00)	2 (25.00)
Metabolic disorders	7 (30.43)	7 (41.78)	-
Respiratory disease	5 (21.74)	1 (6.67)	4 (50.00)
No previous medical history	3 (13.03)	2 (13.33)	1 (12.50)
Missing	3 (11.54)	2 (11.76)	1 (11.11)
Performance status, n (%)			
0	14 (56.00)	9 (56.25)	5 (55.56)
1	11 (44.00)	7 (43.75)	4 (44.44)
Missing	1 (3.85)	1 (5.88)	-
Left Ventricular Ejection Fraction, mean (SD)	65 (6.72)	66.21 (4.92)	62.57 (9.38)
Malnutrition, n (%)	10 (38.46)	5 (29.41)	5 (55.56)
Histology, n (%)			
Squamous cell	1 (3.85)	1 (5.88)	-
Adenocarcinoma	25 (96.15)	16 (94.17)	9 (100)
Stage IV disease *, n (%)	26 (100)	17 (100)	9 (100)
Metastatic sites, n (%)			
Lung	14 (53.85)	9 (52.94)	5 (55.56)
Bone	13 (50.00)	8 (47.05)	5 (55.56)
Brain	9 (34.61)	7 (41.18)	2 (22.22)
Adrenal glands	7 (26.92)	4 (23.53)	3 (33.33)
Liver	3 (11.54)	3 (17.65)	-
Other	2 (7.69)	2 (11.76)	-
Treatment, n (%)			
Carboplatin/Pemetrexed/Pembrolizumab	17 (65.38)	8 (47.05)	9 (100)
Cisplatin/Pemetrexed/Pembrolizumab	7 (26.92)	7 (41.18)	-
Carboplatin/Paclicaxel/Pembrolizumab	2 (7.69)	2 (11.76)	-

* Metastatic status established by multidisciplinary tumor board decision: Overall n = 3; Exercise group n = 2; Control group.

**Table 2:** Changes in anthropometric measures, physical fitness, physical activity level and body composition in the ERICA trial from baseline to M3.

	Exercise Group (n = 17)			Control Group (n = 9)		
	Baseline	M3	*p* Value	Baseline	M3	*p* Value
**Anthropometrics**						
Weight, mean (SD)	75.82 (17.66)	75.19 (19.02)	0.77	64.59 (12.56)	67.52 (14.69)	0.82
Body mass index, mean (SD)	25.17 (3.95)	25.13 (4.29)	0.85	21.83 (3.57)	22.32 (4.02)	0.98
**Physical fitness**						
Isometric quadriceps strength, mean (SD)	329.79 (102.92)	324.25 (88.07)	0.37	247.22 (90.21)	241.62 (135.30)	0.51
Missing, n (%)	3 (17.65)	4 (25.00)		-	1 (12.50)	
Handgrip strength, mean (SD)						
Left side	37.09 (8.37)	37.00 (10.42)	0.97	28.06 (5.09)	26.40 (6.56)	0.44
Missing, n (%)	4 (23.53)	6 (35.29)		-	2 (28.57)	
Right side	37.25 (8.65)	39.02 (9.81)	0.97	26.57 (4.62)	26.14 (7.56)	0.89
Missing, n (%)	3 (17.65)	4 (23.53)		-	2 (28.57)	
VO2 submaximal, mean (SD)	16.16 (4.12)	14.97 (5.31)	0.24	14.25 (3.52)	13.91 (2.96)	0.92
**Leisure-Time Physical Activity**						
Global score, mean (SD)	13.81 (14.46)	30.93 (14.78)	0.06	16.89 (15.89)	23.38 (30.53)	0.60
Active, n, (%)	2 (11.76)	8 (57.14)		2 (22.22)	1 (12.50)	
Moderately Active, n (%)	4 (23.53)	3 (21.43)		2 (22.22)	4 (50.00)	
Insufficiently Active/Sedentary, n (%)	11 (64.71)	3 (21.43)		5 (55.56)	3 (37.50)	
**Body composition**						
Skeletal Muscle Index (cm^2^/m^2^), mean (SD)	49.2 (6.16)	46.2 (7.26)	0.02	45.8 (8.66)	41.4 (9.51)	0.08
Lean Body Mass (kg), mean (SD)	49.2 (6.16)	47.7 (10.3)	0.11	50.4 (9.90)	41.8 (8.08)	0.08
Skeletal Muscle Density (HU), mean (SD)	32.9 (5.90)	35.9 (8.64)	0.99	32.9 (5.90)	36.2 (7.36)	0.55
Subcutaneous Adipose Tissue (cm^2^), mean (SD)	157 (57.5)	154 (55.9)	0.85	137 (152)	140 (154)	0.84
Visceral Adipose Tissue (cm^2^), mean (SD)	200 (133)	188 (116)	0.64	99.8 (46.6)	140 (154)	0.38
Sarcopenia, n (%)	5 (29.4)	9 (56.25)	0.13	4 (44.4)	5 (55.6)	1.00
Missing, n (%)		1 (5.9)			-	

## Data Availability

The raw data supporting the conclusions of this article will be made available by the authors without undue reservation.

## Supplementary Materials

The following supporting information can be downloaded at: https://www.mdpi.com/article/10.3390/jcm15010334/s1, Supplementary Information S1 (SI.1): Feasibility of the exercise intervention in the ERICA trial (n = 17); Supplementary Information S2 (SI.2): Body composition characteristics assessed by third lumbar vertebra (L3) at baseline (D0) by group.; Supplementary Information S3. (SI.3): Body composition characteristics assessed by third lumbar vertebra (L3) at 3-Months (M3) by group.; Supplementary Information S4. (SI.4): Evolution of Patient’s Reported Outcome from Baseline (D0) and 3-month (M3); Supplementary Information S5. (SI.5): Mean difference in group and in between-group change in Patient-Reported Outcomes after 6 months of follow-up (mean, 95% confidence interval); Supplementary Information S6. (SI.6): Treatment completion and adverse events; Supplementary Information S7. (SI.7): Changes in physical fitness from baseline to M3 (n = 25) with 95% confidence intervals.
