# Supplementary material for: Feasibility and Preliminary Efficacy of Aerobic Acute Exercise Prior to Immunotherapy and Chemotherapy Infusion in Patients with Metastatic Non-Small Cell Lung Cancer: A Randomized Controlled Trial
Source: J Clin Med. 2026 Jan 1;15(1):334. doi: 10.3390/jcm15010334 (PMC12787285; doi:10.3390/jcm15010334)
Supplement: Supplementary file 1 [file jcm-15-00334-s001.zip › jcm-4061467-supplementary.pdf]

Supplementary information

Feasibility and preliminary efficacy of aerobic acute exercise prior to immunotherapy and chemotherapy infusion in metastatic non-small cell lung cancer: a randomized controlled trial

Manon Gouez[1,2,3], Olivia Pérol[1,3], Vincent Pialoux[2], Virginie Avrillon[4], Maxime Boussageon[4], Chantal Decroisette[4], Lidia Delrieu[5,6,7], Baptiste Fournier[1], Houssein El Hajj[1], Romane Gille[4], Mathilde His[1,3,] Bénédicte Mastroianni[4], Aurélie Swalduz[4], Maurice Pérol[4], Béatrice Fervers[1,3]

[1]Department of Cancer Prevention and Environment, Léon Bérard Cancer Center, Lyon, France.
[2]Inter-University Laboratory of Human Movement Biology EA7424, University Claude Bernard Lyon 1, University of Lyon, Villeurbanne, France.
[3]Institut National de la Santé et de la Recherche Médicale (INSERM), 1296 Unit Radiations: Defense, Health and Environment, Lyon, France.
[4]Department of Medical Oncology, Léon Bérard Cancer Center, Lyon, France.
[5]Residual Tumor & Response to Treatment Laboratory, RT2Lab, Translational Research Department, INSERM, U932 Immunity and Cancer, Institut Curie, Paris, France.
[6]IRMES, Institute for Research in bioMedicine and Epidemiology of Sport, UPR7329, Université Paris Cité, Paris, France
[7]INSEP, Institut National du Sport, de l'Expertise et de la Performance, Paris, France

Correspondence to: Manon Gouez, Department of Prevention Cancer Environment, Centre Léon Bérard, 28 Prom. Léa et Napoléon Bullukian, 69008 Lyon, France. Email address: manon.gouez@lyon.unicancer.fr

Supplementary Information 1 (SI.1). Feasibility of the exercise intervention in the ERICA trial (n=17). Delay < 30 minutes from ICT perfusion; LI = Light Intensity; MI = Moderate Intensity

	Overall	**Cure 1** *n=17*	**Cure 2** *n=17*	**Cure 3** *n=16*	**Cure 4** *n=16*
**ACUTE AEROBIC EXERCISE**					
**Number of sessions performed**, n (%)	54 (81.82)	12 (70.59)	14 (82.35)	15 (93.75)	13 (81.25)
**Full session completed**, n (%)	28 (51.85)	6 (50.00)	8 (57.14)	8 (53.33)	6 (46.15)
**Dose modification**, n (%)	26 (48.14)	6 (50.00)	6 (42.85)	7 (46.67)	7 (53.85)
Duration reduced, n (%)	15 (27.78)	6 (85.71)	4 (66.66)	4 (57.14)	2 (20.00)
**Not performed**, n (%)	12 (18.18)	5 (29.41)	3 (17.65)	1 (6.25)	3 (18.75)
**Delay from end of exercise to treatment start (min)**, median (IQR)	38.00 [20.00; 60.00]	33.00 [20.00; 51.75]	40.00 [28.00; 58.00]	28.00 [16.00; 41.00]	57.00 [37.00; 65.00]
Delay < 30 minutes, n (%)	20 (37.04)	6 (46.15)	4 (30.77)	8 (61.54)	2 (15.38)
**HOME-BASE WALKING PROGRAM**					
Daily steps counts /week, median (IQR)		8596 [5887; 10 829]	7927 [6741; 12 172]	8539 [6129; 9563]	8529 [6409; 8955]
Achievement of target number of steps, n (%)		7 (63.64)	10 (76.92)	11 (84.62)	9 (100.00)
Missing, n (%)		5 (29.00)	4 (23.53)	4 (23.53)	8 (47.06)

Supplementary Information 2 (SI.2). Body composition characteristics assessed by third lumbar vertebra (L3) at baseline (D0) by group. (A) Skeletal Muscle Density (SMD). **(B)** Skeletal Muscle Index (SMI). **(C)** Lean Body Mass (LBM). **(D)** Body Mass Index (BMI). **(E)** Subcutaneous Adipose Tissue (SAT). **(F)** Visceral Adipose Tissue (VAT). **(G)** Distribution of sarcopenia defined based on L3 SMI index cut-offs <43.0 cm^2/m^2 for men with a BMI <25.0 kg/m^2, <53.0 cm^2/m^2 for men with a BMI ≥ 25.0 kg/m^2, and <41.0 cm^2/m^2 for women (Martin et al. 2013).

Supplementary Information 3. (SI.3) Body composition characteristics assessed by third lumbar vertebra (L3) at 3-Months (M3) by group. (A) Skeletal Muscle Density (SMD). **(B)** Skeletal Muscle Index (SMI). **(C)** Lean Body Mass (LBM). **(D)** Body Mass Index (BMI). **(E)** Subcutaneous Adipose Tissue (SAT). **(F)** Visceral Adipose Tissue (VAT). **(G)** Distribution of sarcopenia defined based on L3 SMI index cut-offs <43.0 cm^2/m^2 for men with a BMI <25.0 kg/m^2, <53.0 cm^2/m^2 for men with a BMI ≥ 25.0 kg/m^2, and <41.0 cm^2/m^2 for women (Martin et al. 2013).

Supplementary Information 4. (SI.4) Evolution of Patient's Reported Outcome from Baseline (D0) and 3-month (M3)

	Exercise group			***Control group***		
	Baseline (n=17)	*M3 (n=14)*	*P*	*Baseline (n=8)*	*M3 (n=7)*	*p*
**EORTC QLQ C30**						
**Global health**	56.77 (20.46)	67.26 (19.47)	0.57	52.78 (17.68)	44.79 (13.32)	0.20
**Function scales (mean, SD)**						
Physical	85.00 (14.30)	87.62 (14.99)	1.00	77.04 (13.38)	80.00 (15.12)	1.00
Role	73.96 (27.87)	71.43 (22.10)	0.51	70.83 (19.42)	58.33 (28.17)	0.10
Emotional	62.22 (18.60)	76.19 (15.97)	0.08	75.00 (16.67)	67.86 (26.97)	0.68
Cognitive	84.52 (17.86)	84.72 (19.41)	0.85	81.48 (22.74)	75.00 (28.17)	0.85
Social	83.33 (22.77)	82.14 (16.62)	0.44	77.78 (20.41)	68.75 (18.77)	0.42
**Lung cancer module (mean, SD)**						
Dyspnoea	13.19 (17.32)	13.49 (15.21)	0.17	22.22 (24.22)	23.61 (20.09)	0.10
Coughing	25.00 (28.54)	19.05 (17.12)	0.57	48.15 (33.79)	37.50 (21.36)	0.85
Haemoptysis	2.08 (8.33)	4.76 (12.10)	1.00	7.41 (14.70)	-	0.35
Sore mouth	2.08 (8.33)	2.38 (8.91)	-	3.70 (11.11)	12.50 (24.80)	0.35
Dysphagia	-	4.76 (12.10)	0.35	7.41 (14.70)	28.57 (48.80)	0.42
Peripheral neuropathy	8.33 (19.25)	11.90 (21.11)	1.00	14.81 (33.79)	20.83 (35.36)	1.00
Alopecia	-	21.43 (30.96)	0.06	11.11 (16.67)	20.83 (30.54)	0.35
Pain in chest	16.67 (21.08)	4.76 (12.10)	0.15	11.11 (16.67)	12.50 (17.25)	1.00
Pain in arm or shoulder	14.58 (20.97)	2.38 (8.91)	0.13	25.93 (27.78)	8.33 (15.43)	0.17
Pain in other parts	23.33 (27.44)	16.67 (26.59)	0.35	20.83 (30.54)	19.05 (32.53)	0.85
**QLQ FA12 (mean, SD)**						
Physical fatigue	32.50 (21.89)	31.43 (20.66)	0.33	31.85 (11.92)	54.17 (25.06)	0.06
Emotional fatigue	32.64 (24.16)	25.40 (23.64)	0.64	32.10 (17.07)	43.06 (23.34)	0.03
Cognitive fatigue	17.71 (21.49)	8.33 (14.25)	0.34	18.52 (25.61)	23.81 (25.20)	0.79
**Insomnia Severity Index (mean, SD)**						
Global score	10.06 (4.97)	7.00 (5.99)	0.02	9.62 (7.25)	8.50 (4.93)	0.48

Supplementary Information 5. (SI.5) Mean difference in group and in between- group change in Patient-Reported Outcomes after 6 months of follow-up (mean, 95% confidence interval)

	Exercise group		Control group		Mean difference	
**EORTC QLQ C30**						
**Global health**	18.95	(-28.11, 9.78)	14.29	(-0.28, 28.85)	-23,45	(-45.39, -1.52)
**Function scales**						
Physical	-13.33	(-39.84, 13.17)	-2.86	(-7.71, 1.99)	-10.48	(-37.11, 16.16)
Role	-11.67	(-44.46, 21.13)	16.67	(-12.85, 46.18)	-28.33	(-68.53, 11.87)
Emotional	-2.50	(-14.44, 9.44)	-5.56	(-18.72, 7.61)	3.06	(-12.82, 18.94)
Cognitive	-9.52	(-21.65, 2.6)	-3.33	(-20.65, 13.98)	-6.19	(-24.4, 12.02)
Social	-16.67	(-42.42, 9.09)	9.52	(-18.42, 37.46)	-26.19	(-60.69, 8.31)
**Lung cancer module**						
Dyspnoea	-2.22	(-11.25, 6.8)	-7.94	(-22.12, 6.24)	5.71	(-9.72, 21.14)
Coughing	-10.00	(-26.09, 6.09)	-9.52	(-32.83, 13.78)	-0.48	(-26.36, 25.41)
Haemoptysis		-		-		
Sore mouth	3.33	(-4.21, 10.87)	9.52	(-5.52, 24.57)	-6.19	(-21.88, 9.5)
Dysphagia	0.00	(-11.24, 11.24)	-5.56	(-39.95, 28.84)	5.56	(-28.84, 39.95)
Peripheral neuropathy	23.33	(-1.93, 48.59)	19.05	(-15.91, 54)	4.29	(-35.06, 43.63)
Alopecia	3.33	(-14.26, 20.93)	-4.76	(-37.72, 28.2)	8.10	(-26.59, 42.78)
Pain in chest	6.67	(-8.41, 21.75)	0.00	(-17.8, 17.8)	6.67	(-14.52, 27.86)
Pain in arm or shoulder	3.33	(-10.2, 16.87)	14.29	(-2.19, 30.76)	-10.96	(-30.33, 8.43)
Pain in other parts	4.76	(-16.51, 26.04)	4.76	(-16.51, 26.04)	0.00	(-26.79, 26.79)
**QLQ FA12**						
Physical fatigue	16.00	(-5.35, 37.35)	-11.43	(-35.23, 12.37)	27.43	(-1.6, 56.46)
Emotional fatigue	5.56	(-21, 32.12)	-9.52	(-23.35, 4.3)	15.08	(-13.15, 43.31)
Cognitive fatigue	16.67	(4.1, 29.24)	0.00	(-22.12, 22.12)	16.67	(-6.45, 39.78)
**Insomnia Severity Index**						
Global score	13.33	(-3.34, 30.01)	3.14	(-1.04, 7.33)	-0.74	(-5.17, 3.69)

Supplementary Information 6. (SI.6) Treatment completion and adverse events

	**Overall** (n=26)	**Exercise group*** (n=17)	**Control group*** (n=9)
**Treatment modification,** n (%)			
Cycle 1	3 (12.00)	3 (17.65)	-
Cycle 2	5 (20.00)	3 (17.65)	2 (25.00)
Cycle 3	9 (36.00)	3 (18.75)	6 (75.00)
Cycle 4	7 (29.17)	4 (25.00)	3 (37.50)
**Adverse event grade ≥ 3,** n (%)			
Grade 3	14 (82.35)	3 (21.42)	11 (78.57)
Grade 4	3 (17.65)	1 (33.33)	2 (66.66)
**Type of adverse event (grade ≥ 3),** n			
Anemia	3	2	1
Anorexia	2	1	1
Aplasia	1	0	1
Asthenia	3	0	3
Neutropenia	2	0	2
Thrombopenia	3	0	3
Weight loss	1	0	1
Diarrhea post chemotherapy	1	0	1
Troponin I increased	2	1	1
**Treatment response at M3**			
Stable disease	7 (28.00)	3 (18.75)	4 (44.44)
Partial response	14 (53.85)	10 (62.50)	4 (44.44)
Disease progression	2 (8.00)	2 (12.50)	-
No. of deaths	2 (8.00)	1 (6.25)	1 (11.11)
Data missing	1 (4.00)	1 (5.90)	-

**Number of patients in the exercise group: Cycle 1-2: n=17; Cycle 3-4: n=16; and in the control group: Cycle 1-3: n=9; Cycle 4:*

Supplementary Information 7. (SI.7) Changes in physical fitness from baseline to M3 (n=25) with 95% confidence intervals
